# The New Light on the Nervous System

**Published:** 1897-06-12

**Authors:** 


### THE NEW LIGHT ON THE NERVOUS SYSTEM.

By A TEACHER OF PHYSIOLOGY.

That the progress of medicine is indissolubly bound up with that of physiology is a proposition which is generally admitted but seldom obvious, for the relations between the two are but rarely insisted on to a sufficient extent. Nor is it often that a new fact in physiology comes to light of such deep importance as to directly influence and alter the standpoint of the corresponding department in medicine. Still, pathology is, after all, only physiology gone wrong, and this is forcibly brought home to us by the changes in neurology which have resulted from the histological researches of Golgi and his successors. It is just ten years since Golgi brought before the scientific world a new weapon in the armamentarium of the histologist, namely, a method of impregnating the cells and cell-processes of the central nervous system with a heavy metal such as silver or mercury, so as to show them up black against a colourless or yellow background of fibres. Since then the progress made by the aid of this method and its modifications has been enormous, particularly in the hands of Van Gehuchten, Lenhossek, and especially Ramon y Cajal.

The first result of this work has been the abandonment of the former ideas with regard to the relationship of nerve-cells and nerve-fibres; the view that the nerve-cell was the centre of "nervous action," and the fibre merely a conducting wire developed from it, is now untenable. In place of this one sees the establishment of a nervous unit, a specialised element the integrity of which is essential to the performance of nervous function. Just as the atom is the unit of physics, the molecule that of chemistry, and the cell of physiology, so the neuron is the unit of neurology. A neuron consists of a single nerve-cell, together with all the processes which are dependent upon it for their nutrition. Of these processes all save one (in rare cases two) are fine, and rapidly break up into a mass of delicate branches; these are known as dendrites. The other process is the axis-cylinder of the nerve-fibre which arises from the cell, and is called, somewhat unnecessarily, the neuraxon, the axis-cylinder neuron giving off at right angles, by the way, fine branches known as collaterals till it terminates in one of the numerous forms of nerve-endings—motor, sensory, or secretory. These nerve-endings are mainly variations of one type, a division of the axis-cylinder into a number of fibrillar branches which ramify in a mass of nucleated protoplasm. The nerve is thus brought into connection with the particular tissue in which it terminates; it should be mentioned that the branching is only apparent, as close examination of an axis-cylinder shows it to consists of a number of parallel fibrils, each in a little sheath of neurokeratin, which leave the nerve-fibre as collaterals, or as the fibrils of the end-organ as the case may be. A neuron, therefore, consists of three parts, a nerve-cell with its ramifying dendrites, an axis-cylinder (neuraxon) and a nerve-ending; each of these is useless without the others, and the whole mechanism of the central nervous system is dependent upon the action of the constituent neurons upon each other, for the whole nervous system consists of neurons imbedded in a neuroglial jelly.

The main interest of all this must obviously lie in the inter-relation of the neurons of the brain and spinal cord. Now a nerve-ending in these organs consists of a brush of fibrils embedded in protoplasm, the neuroglia. These fibrils terminate in minute knobs, as also do the branches of the dendrites. The two sets of knobs, from nerve-endings and dendrites, are in close relation to each other, but do not, as was formerly believed, come into actual contact; they interlace like the branches and leaves of the trees in a forest. The dendrites are undoubtedly nervous in function, though Golgi at first believed them to merely suck nutriment up from the neuroglia. Here, then, is the key to nervous action. It is a process of induction spreading across the minute space separating the nerve-endings of one neuron from the dendrites of another. Thus the impulse transmitted down to the former induces another impulse, similar or modified as the case may be, in the latter. The cell therefore plays no part in the true inwardness of nervous activity; the seat of this is the meshwork of neuronic fibrils in the grey matter, and the nerve-cell subserves merely the nutrition of its neuron.

The value, one may even say the beauty, of this work is seen in its application to the physiology and pathology of the central nervous system. Take, for example, a simple reflex action. Here all we have to assume is the existence of two neurons, a sensory and a motor; the afferent impulse passes up to the central termination of the former and there induces an efferent impulse in the dendrites of the latter. When the stimulus is more powerful the inductive force is greater and other sets of dendrites are affected, leading to associated action. Here is a complex and baffling problem solved almost at a stroke of the pen.

It is hardly necessary to say that much valuable work has been done of late in tracing out the neurons of the various tracts of the spinal cord and, so far with less precision, of the brain. It must suffice to give some examples of the light thrown by such work upon nervous diseases. Thus it has been shown that a motor impulse in its passage from cortex to muscle need only traverse two neurons, one having its nerve-cell and dendrites in the motor cortex, its axis-cylinder in the pyramidal tract (from corona radiata downwards), and its nerve-ending in the grey matter of the anterior cornu of the spinal cord, the other having its cell and dendrites in this anterior cornu, its axis-cylinder in a peripheral nerve, and its termination in a motor end-plate. From the pathological point of view, either of these neurons may be affected separately, and each one may suffer centripetally and centrifugally. Thus peripheral neuritis is a centripetal disease of the lower neuron; lateral sclerosis, which appears to actually begin in the nerve-endings, between and around the ganglion-cells of the anterior horn, a similar affection of the upper. Secondary degeneration in the lateral columns after hemiplegia or cortical trauma is a morbid process spreading down the upper neuron; progressive muscular atrophy and acute anterior poliomyelitis affect the lower segment in like manner. Each of these diseases is notably limited to its own neuron, excellent evidence, if any were needed, of the absence of direct contact between the dendrites and nerve-endings. The nutritive function of the cells is well shown by the fact that nerve and muscle-atrophy only arise, as a rule, from destruction of the cells. With regard to diseases of the sensory neurons, tabes may be cited; this affects the whole neuron connected with the afferent nerves of the muscles, and having its cell-station in the posterior root ganglion. Instances such as these must be enough to convince anyone of the value of the new method of investigation in the systematisation of nervous diseases; to its future usefulness in the elucidation of still obscure affections no limit can as yet be set.

